# Long‐term efficacy of tafamidis in patients with transthyretin amyloid cardiomyopathy by National Amyloidosis Centre stage

**DOI:** 10.1002/ejhf.3696

**Published:** 2025-06-09

**Authors:** Thibaud Damy, Rong Wang, Mathew S. Maurer, Julian D. Gillmore, Marianna Fontana

**Affiliations:** ^1^ Department of Cardiology Henri Mondor University Hospital Créteil France; ^2^ Pfizer Inc Groton CT USA; ^3^ Division of Cardiology Columbia University Irving Medical Center New York NY USA; ^4^ National Amyloidosis Centre, Division of Medicine University College London London UK

**Keywords:** Transthyretin, Amyloidosis, ATTR‐CM staging, Cardiomyopathy

## Abstract

**Aims:**

Tafamidis is an approved treatment for patients with transthyretin amyloid cardiomyopathy (ATTR‐CM) based on the 30‐month Tafamidis in Transthyretin Cardiomyopathy Clinical Trial (ATTR‐ACT). This post‐hoc analysis evaluated outcomes in ATTR‐ACT and its long‐term extension study (LTE) by baseline National Amyloidosis Centre (NAC) stage.

**Methods and results:**

Patients received either the approved dose of tafamidis 80 mg or placebo in ATTR‐ACT and tafamidis in the LTE. All‐cause and cardiovascular (CV)‐related mortality, CV‐related hospitalizations, and Kansas City Cardiomyopathy Questionnaire overall summary and clinical summary (KCCQ‐OS/CS) scores were assessed up to 90 months of follow‐up. Of 353 patients, 350 were evaluable for NAC staging. At baseline, 42%, 38%, and 20% were NAC stage I, II, and III, respectively. At the end of study, all‐cause mortality was lower in the continuous tafamidis versus placebo to tafamidis groups at NAC stages I (36% vs. 61%; hazard ratio [HR] 0.43, *p* < 0.001) and II (55% vs. 74%; HR 0.51, *p* = 0.003); with a numerical trend at stage III (69% vs. 88%; HR 0.75, *p* = 0.298). Survival curves diverged early in patients at NAC stage I, but later at higher stages. Similar patterns were observed for CV‐related mortality. Continuous tafamidis versus placebo to tafamidis groups at NAC stages I/II had lower CV‐related hospitalization rates and frequently smaller declines in KCCQ‐OS/CS scores over follow‐up; with favourable trends at stage III.

**Conclusions:**

Tafamidis treatment reduced the risk of mortality and hospitalization in patients with NAC stages I/II ATTR‐CM, with favourable trends at stage III. This illustrates the importance of early diagnosis and initiation of disease‐modifying therapy.

Clinical Trial Registration: ClinicalTrials.gov, NCT01994889, NCT02791230.

## Introduction

Transthyretin (TTR) amyloid cardiomyopathy (ATTR‐CM) is an underdiagnosed and progressive disease caused by the deposition of variant or wild‐type TTR amyloid in the myocardium, leading to heart failure (HF).^1^, ^2^, ^3^ The median survival from diagnosis of untreated patients was reported to be approximately 2–6 years.^2^, ^3^

Stratification of patients' prognoses is a key step to optimize management of patients both in clinical practice and for clinical trials.^4^, ^5^ Current ATTR‐CM staging systems use a combination of cardiac biomarkers (e.g. N‐terminal pro‐B‐type natriuretic peptide [NT‐proBNP] and troponin T), New York Heart Association (NYHA) functional class, loop diuretic dose, and renal function.^2^, ^4^, ^5^, ^6^, ^7^ The most widely used National Amyloidosis Centre (NAC) staging system stratifies patients with wild‐type and variant ATTR‐CM into three categories based on their NT‐proBNP concentration (>3000 or ≤3000 ng/L) and estimated glomerular filtration rate (eGFR; ≥45 or <45 ml/min/1.73 m^2^).^2^ A recent study proposed an expanded NAC staging system to include a new stage IV category with a high 1‐year mortality risk, defined as patients with NT‐proBNP ≥10 000 ng/L, irrespective of eGFR.^8^

Tafamidis meglumine 80 mg and the bioequivalent tafamidis free acid 61 mg is an approved treatment for patients with ATTR‐CM.^9^ The approval was supported by findings from the pivotal phase 3 Tafamidis in Transthyretin Cardiomyopathy Clinical Trial (ATTR‐ACT; NCT01994889).^10^ Patients treated with tafamidis versus placebo showed 30% lower risk of all‐cause mortality, and lower rate of decline in functional capacity and quality of life over 30 months of treatment, and 32% reduced annual risk of cardiovascular (CV)‐related hospitalizations.^10^ After completing ATTR‐ACT, a long‐term extension study (LTE; NCT02791230) enabled patients to receive tafamidis for an additional 60 months or until commercially available in their region. Findings from the LTE demonstrated that patients initially treated with tafamidis in ATTR‐ACT had significantly better survival than those first treated with placebo, emphasizing the clinical benefit of early diagnosis and treatment of patients with ATTR‐CM.^11^, ^12^

We present the results of a post‐hoc analysis using data from ATTR‐ACT and the LTE, comparing long‐term survival, CV‐related hospitalizations, and changes in clinical symptoms and quality of life by NAC stage at ATTR‐ACT baseline. Patients received either continuous tafamidis at the approved dose (meglumine 80 mg/free acid 61 mg) in both studies, or placebo in ATTR‐ACT and tafamidis in the LTE.

## Methods

### Studies

**Figure 1:**
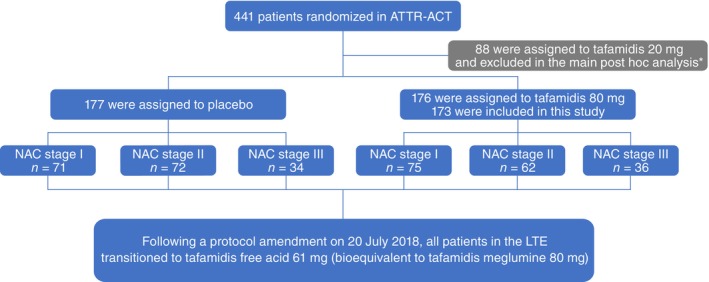
Patients in the Tafamidis in Transthyretin Cardiomyopathy Clinical Trial (ATTR‐ACT) and the long‐term extension study (LTE) included in this post‐hoc analysis. NAC, National Amyloidosis Centre. *Tafamidis meglumine 20 mg is not an approved treatment for patients with transthyretin amyloid cardiomyopathy. In this study, the main analysis included patients who received the approved dose of tafamidis meglumine 80 mg or placebo in ATTR‐ACT and tafamidis in the LTE. The sensitivity and supplementary analyses included patients who received tafamidis 80/20 mg (pooled) or placebo in ATTR‐ACT and tafamidis in the LTE. Of 176 patients randomized to tafamidis meglumine 80 mg in ATTR‐ACT, three did not have both a baseline estimated glomerular filtration rate and N‐terminal pro‐B‐type natriuretic peptide concentration to allow NAC staging.

ATTR‐ACT was a multicentre, international, double‐blind, placebo‐controlled, parallel‐design, randomized, phase 3 trial of tafamidis in patients with ATTR‐CM.^10^ Patients (*n* = 441) were randomized 2:1:2 to receive tafamidis meglumine 80 mg or 20 mg (*n* = 264, pooled), or placebo (*n* = 177) for 30 months, stratified by *TTR* genotype (wild‐type or variant) and NYHA class (I or II/III) (*Figure* *1*). After completing ATTR‐ACT, patients were eligible to join the LTE and received tafamidis for up to 60 months. Patients receiving tafamidis meglumine (80 or 20 mg) in ATTR‐ACT initially continued this dose in the LTE. Patients receiving placebo in ATTR‐ACT were randomized 2:1 to receive tafamidis 80 or 20 mg, stratified by *TTR* genotype, in the LTE.^11^ Following a protocol amendment on 20 July 2018, all patients in the LTE transitioned to the approved once‐daily tafamidis free acid 61 mg.

Both ATTR‐ACT and the LTE were conducted in accordance with ethical principles derived from the Declaration of Helsinki and the International Council for Harmonization Good Clinical Practice guidelines. The studies were approved by the ethics committees or institutional review boards at each participating site. All patients provided written informed consent.^10^, ^11^

### Patients

Patients (aged 18–90 years) were eligible to participate in ATTR‐ACT if they had wild‐type or variant ATTR‐CM confirmed by the presence of amyloid deposits in biopsy specimens, a history of HF with ≥1 prior hospitalization due to HF or clinical symptoms associated with HF, an end‐diastolic intraventricular septal wall thickness >12 mm as assessed by echocardiography, a 6‐min walk test distance >100 m, and an NT‐proBNP concentration ≥600 ng/L.^10^, ^11^ Patients were not included if they had an eGFR <25 ml/min/1.73 m^2^, modified body mass index <600, or NYHA functional class IV HF; or had light‐chain amyloidosis or HF that was not due to ATTR‐CM, a history of liver or heart transplantation, or an implanted cardiac assist device.

### Outcomes

The standard NAC staging system categorizes patients into three stages: I (NT‐proBNP ≤3000 ng/L and eGFR ≥45 ml/min/1.73 m^2^), II (NT‐proBNP >3000 ng/L or eGFR <45 ml/min/1.73 m^2^), and III (NT‐proBNP >3000 ng/L and eGFR <45 ml/min/1.73 m^2^).^2^ Patients in the continuous tafamidis group received tafamidis in ATTR‐ACT and tafamidis in the LTE. Patients in the placebo to tafamidis group received placebo in ATTR‐ACT and transitioned to tafamidis in the LTE. In this post‐hoc analysis, the key outcomes were all‐cause and CV‐related mortality throughout ATTR‐ACT and the LTE by NAC stages I–III (defined based on baseline values in ATTR‐ACT). Supportive outcomes included the proportion of patients with CV‐related hospitalizations, total annual rate of CV‐related hospitalizations among all patients, and least square mean change from baseline in Kansas City Cardiomyopathy Questionnaire (KCCQ) clinical summary (KCCQ‐CS) and overall summary (KCCQ‐OS) scores. The main post‐hoc analysis only included patients who received the approved tafamidis meglumine 80 mg dose or placebo in ATTR‐ACT, categorized using the traditional NAC staging system (I–III). Moreover, the expanded NAC staging system (I–IV) was used in supplemental analyses (online supplementary *Appendix* *S1*).

### Statistical analysis

Data from patients who received tafamidis meglumine 20 mg in ATTR‐ACT were not included in the main analysis, as this dose has not been approved for the treatment of patients with ATTR‐CM. Neither ATTR‐ACT nor its LTE was statistically powered to address the impact of tafamidis by NAC stage; therefore, the results are exploratory in nature. Further sensitivity and supplemental analyses were conducted (online supplementary *Appendix* *S1*). Statistical analysis was done using SAS version 9.4 (SAS Institute Inc, Cary, NC, USA). Statistical significance was defined as *p* ≤ 0.05.

Cardiovascular‐related mortality and hospitalizations were adjudicated by an external committee blinded to treatment in ATTR‐ACT. In the LTE, an algorithm was used to determine the causality of death using data collected in electronic data records by the investigator. Deaths due to ATTR‐CM were considered CV‐related. In other cases, relatedness to pre‐specified preferred terms was used to confirm CV‐related causality (online supplementary *Table* *S1*). Hospitalization was defined as non‐elective admission to an acute care setting for medical therapy for ≥24 h. CV‐related hospitalizations included those with a discharge diagnosis indicating a CV‐related reason (using algorithmic process detailed above) and that lead to a serious adverse event (online supplementary *Table* *S1*).

All‐cause or CV‐related mortality between treatment groups was assessed by a Cox proportional hazards model adjusted for baseline *TTR* genotype (wild‐type or variant) and visualized using Kaplan–Meier curves. A *p* for interaction analysis using the Wald *χ* ^2^ test was conducted to assess interaction between treatment group and NAC stage for all‐cause and CV‐related mortality from Cox proportional hazards model. Heart transplant and cardiac mechanical assist device implantation were treated as death events in the mortality assessments. The relative risk of CV‐related hospitalizations was compared using a Poisson regression model with treatment as the main effect and adjusted for baseline *TTR* genotype as covariate.

The KCCQ‐OS and ‐CS scores are patient‐reported measures that assess quality of life, and total symptoms and physical limitations associated with HF, respectively. A change of 5 points in the KCCQ score is considered small but clinically important, whereas changes of 10 and 20 points are considered moderate to large, and large to very large, respectively.^13^ In this study, KCCQ‐OS and ‐CS scores were assessed using a mixed model for repeated measures and analysis of covariance, with an unstructured matrix. Treatment, visit, *TTR* genotype, and visit‐by‐treatment interaction were treated as fixed effects.

## Results

### Patient demographics

Overall, 350 of 353 patients who received tafamidis 80 mg or placebo in ATTR‐ACT had evaluable data for NAC staging. Of the 350 patients, most were male (89%, *n* = 312), White (80%, *n* = 279), carried a wild‐type *TTR* genotype (76%, *n* = 265), and with NYHA functional class II HF symptoms (59%, *n* = 206). At ATTR‐ACT baseline, 42% (*n* = 146), 38% (*n* = 134), and 20% (*n* = 70) had NAC stage I, II, and III ATTR‐CM, respectively (*Table* *1*).

**Table 1:** Baseline characteristics of patients by National Amyloidosis Centre stages I–III

	NAC stage I (*n* = 146)		NAC stage II (*n* = 134)		NAC stage III (*n* = 70)	
	Placebo to tafamidis (*n* = 71)	Continuous tafamidis^a^ (*n* = 75)	Placebo to tafamidis (*n* = 72)	Continuous tafamidis^a^ (*n* = 62)	Placebo to tafamidis (*n* = 34)	Continuous tafamidis^a^ (*n* = 36)
Age, years, mean (SD)	72.1 (7.4)	73.2 (7.4)	74.6 (5.7)	75.4 (6.9)	77.0 (6.0)	79.1 (6.1)
Sex, *n* (%)						
Male	62 (87.3)	69 (92.0)	66 (91.7)	58 (93.5)	29 (85.3)	28 (77.8)
Female	9 (12.7)	6 (8.0)	6 (8.3)	4 (6.5)	5 (14.7)	8 (22.2)
Race, *n* (%)						
White	61 (85.9)	59 (78.7)	61 (84.7)	53 (85.5)	24 (70.6)	21 (58.3)
Black	9 (12.7)	10 (13.3)	9 (12.5)	5 (8.1)	8 (23.5)	11 (30.6)
Asian	1 (1.4)	5 (6.7)	2 (2.8)	4 (6.5)	2 (5.9)	2 (5.6)
American Indian or Alaska Native	0	1 (1.3)	0	0	0	2 (5.6)
*TTR* genotype, *n* (%)						
Wild‐type	52 (73.2)	59 (78.7)	57 (79.2)	51 (82.3)	25 (73.5)	21 (58.3)
Variant	19 (26.8)	16 (21.3)	15 (20.8)	11 (17.7)	9 (26.5)	15 (41.7)
NT‐proBNP, ng/L, mean (SD)	1837.3 (665.5)	1762.7 (694.3)	4533.9 (2717.8)	4937.3 (3087.3)	6580.5 (3583.1)	6680.5 (3094.9)
mBMI, mean (SD)^b^	1094 (197.7)	1089 (173.7)	1019 (185.6)	1037 (159.8)	1110 (190.1)	1049 (184.1)
eGFR, ml/min/1.73 m^2^, mean (SD)^c^	66.0 (13.4)	67.8 (13.2)	54.8 (14.0)	56.0 (11.9)	35.3 (6.3)	34.8 (6.6)
Troponin I, ng/ml, mean (SD)	0.1 (0.2)	0.1 (0.1)	0.2 (0.2)^d^	0.2 (0.1)	0.2 (0.2)	0.7 (2.0)
NYHA class, *n* (%)						
I	6 (8.5)	11 (14.7)	6 (8.3)	3 (4.8)	1 (2.9)	1 (2.8)
II	48 (67.6)	53 (70.7)	39 (54.2)	38 (61.3)	14 (41.2)	14 (38.9)
III	17 (23.9)	11 (14.7)	27 (37.5)	21 (33.9)	19 (55.9)	21 (58.3)
Follow‐up duration, months, median (95% CI)^e^	64.2 (57.5–77.8)	63.3 (59.9–68.8)	59.1 (51.6–81.5)	61.0 (58.1–76.0)	53.4 (50.2–NE)	51.6 (31.6–60.9)

BMI, body mass index; CI, confidence interval; eGFR, estimated glomerular filtration rate; mBMI, modified body mass index; NAC, National Amyloidosis Centre; NE, non‐estimable; NT‐proBNP, N‐terminal pro‐B‐type natriuretic peptide; NYHA, New York Heart Association; SD, standard deviation; *TTR*, transthyretin.

^a^ Following a protocol amendment, all patients in the long‐term extension study transitioned to tafamidis free acid 61 mg (bioequivalent to tafamidis meglumine 80 mg).

^b^ mBMI was calculated as the serum albumin level (g/L) multiplied by the BMI (weight in kg/square of the height in meters).

^c^ eGFR was estimated using the Chronic Kidney Disease Epidemiology Collaboration equation.

^d^ *n* = 71.

^e^ Calculated using the Kaplan–Meier method.

Overall, patients at NAC stage III tended to be older (mean age [standard deviation, SD]: 78.0 [6.1] years) vs. stage II (75.0 [6.3] years) and stage I (72.7 [7.4] years), more likely female (19% vs. 7% and 10%), with a variant *TTR* genotype (34% vs. 19% and 24%), and with NYHA class III HF (57% vs. 36% and 19%). At study completion, the median follow‐up duration was 52 (continuous tafamidis) and 53 months (placebo to tafamidis) in patients with baseline NAC stage III. The respective median follow‐up duration in patients for each treatment group with baseline NAC stages II and I were 61 and 59 months, and 63 and 64 months.

### All‐cause mortality

In the Cox proportional hazards model for all‐cause mortality, no significant interaction was observed between treatment and NAC stage at the end of study (EOS, *p* for interaction = 0.575), at month 60 (*p* for interaction = 0.442), and at month 30 (*p* for interaction = 0.530). This indicated that survival was significantly different across NAC stages regardless of treatment. Therefore, subgroup survival analysis was performed to compare treatment effects within each NAC stage.

**Figure 2:**
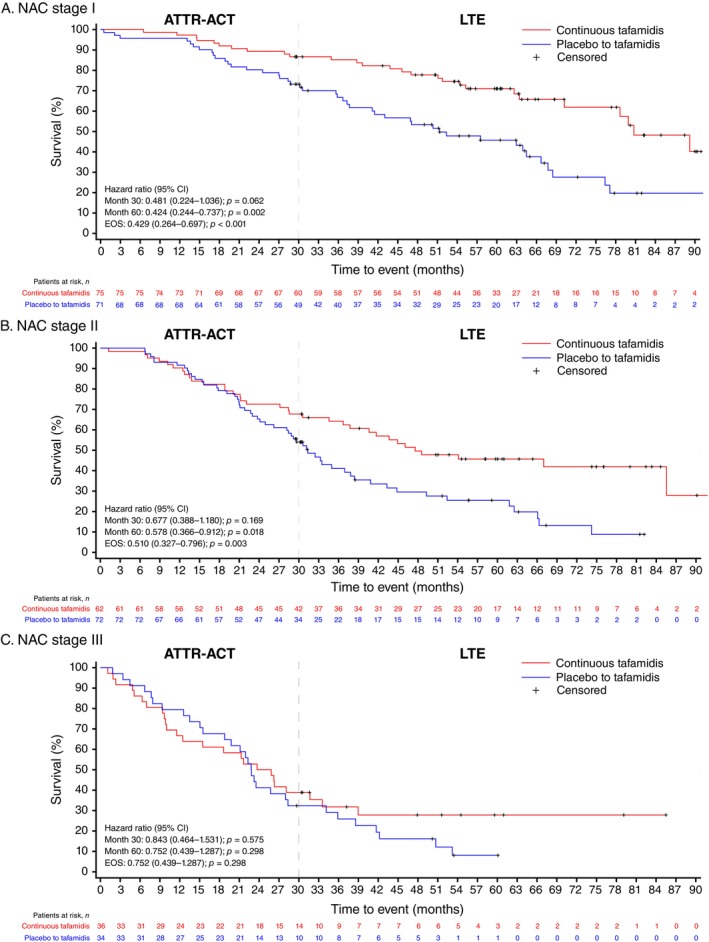
Kaplan–Meier curves of all‐cause mortality in patients with baseline National Amyloidosis Centre (NAC) stage I–III transthyretin amyloid cardiomyopathy. ATTR‐ACT, Tafamidis in Transthyretin Cardiomyopathy Clinical Trial; CI, confidence interval; EOS, end of study; LTE, long‐term extension study.

At the end of follow‐up, the proportions with all‐cause mortality in the continuous tafamidis versus placebo to tafamidis groups were 36% versus 61%, 55% versus 74%, and 69% versus 88% for patients with baseline NAC stage I, II, and III, respectively (*Table* *2*). The risk of all‐cause mortality until the EOS was significantly lower (*p* < 0.05) in the continuous tafamidis versus placebo to tafamidis groups across NAC stages I (hazard ratio [HR], 95% confidence interval [CI]: 0.43 [0.26–0.70]; *p* < 0.001) and II (0.51 [0.33–0.80]; *p* = 0.003), with a numerical trend observed in stage III (0.75 [0.44–1.29]; *p* = 0.298) (*Table* *2*; Graphical Abstract). Kaplan–Meier all‐cause survival curves showed that continuous tafamidis treatment reduced the risk of all‐cause mortality, with curves diverging early in patients at baseline NAC stage I (*Figure* *2*). Divergence emerged later in patients at higher NAC stages. At month 60, the proportions with all‐cause mortality in the continuous tafamidis versus placebo to tafamidis groups were 27% versus 49% (HR [95% CI]: 0.42 [0.24–0.74]; *p* = 0.002), 52% versus 68% (0.58 [0.37–0.91]; *p* = 0.018), and 69% versus 88% (0.75 [0.44–1.29]; *p* = 0.298) for patients at NAC stages I, II, and III, respectively. Generally, patients with a higher NAC stage had shorter survival. Within NAC groups, median survival time (95% CI) was numerically longer in patients treated with continuous tafamidis versus placebo to tafamidis (I: 81 [70–non‐estimable (NE)] vs. 51 [38–67] months; II: 48 [37–NE] vs. 31 [26–38] months; and III: 25 [12–34] vs. 23 [16–28] months) (*Table* *2*).

**Table 2:** All‐cause and cardiovascular‐related mortality and cardiovascular‐related hospitalization in the Tafamidis in Transthyretin Cardiomyopathy Clinical Trial and the long‐term extension study across baseline National Amyloidosis Centre stages I–III until the end of study

	NAC stage I (*n* = 146)		NAC stage II (*n* = 134)		NAC stage III (*n* = 70)	
	Placebo to tafamidis (*n* = 71)	Continuous tafamidis^a^ (*n* = 75)	Placebo to tafamidis (*n* = 72)	Continuous tafamidis (*n* = 62)	Placebo to tafamidis (*n* = 34)	Continuous tafamidis (*n* = 36)
All‐cause mortality, *n* (%)^a^	43 (60.6)	27 (36.0)	53 (73.6)	34 (54.8)	30 (88.2)	25 (69.4)
Death	40 (56.3)	23 (30.7)	51 (70.8)	31 (50.0)	29 (85.3)	23 (63.9)
Heart transplant	3 (4.2)	4 (5.3)	2 (2.8)	3 (4.8)	1 (2.9)	0
CMAD	0	0	0	0	0	2 (5.6)
KM estimate of time to event, months, median (95% CI)	51.3 (37.7–66.7)	80.8 (70.2–NE)	31.3 (26.2–37.8)	47.6 (36.7–NE)	22.8 (15.5–28.4)	24.8 (11.5–33.5)
Hazard ratio for continuous tafamidis vs. placebo to tafamidis (95% CI)^b^	0.429 (0.264–0.697)		0.510 (0.327–0.796)		0.752 (0.439–1.287)	
*p*‐value^b^	<0.001		0.003		0.298	
CV‐related mortality, *n* (%)^a^	33 (46.5)	19 (25.3)	42 (58.3)	26 (41.9)	26 (76.5)	22 (61.1)
Death	30 (42.3)	15 (20.0)	40 (55.6)	23 (37.1)	25 (73.5)	20 (55.6)
Heart transplant	3 (4.2)	4 (5.3)	2 (2.8)	3 (4.8)	1 (2.9)	0
CMAD	0	0	0	0	0	2 (5.6)
KM estimate of time to event, months, median (95% CI)	62.9 (43.0–NE)	NE (78.7–NE)	35.0 (29.7–44.8)	NE (43.6–NE)	23.2 (19.8–34.1)	26.2 (15.5–39.0)
Hazard ratio for continuous tafamidis vs. placebo to tafamidis (95% CI)^b^	0.394 (0.223–0.697)		0.513 (0.311–0.848)		0.747 (0.420–1.328)	
*p*‐value^b^	0.001		0.009		0.320	
CV‐related hospitalization						
Patients with ≥1 CV‐related hospitalization, *n* (%)	23 (32.4)	29 (38.7)	15 (20.8)	19 (30.6)	5 (14.7)	6 (16.7)
Total annual rate among all patients^c^	0.31	0.19	0.29	0.14	0.23	0.13
Risk ratio for continuous tafamidis vs. placebo to tafamidis (95% CI)^d^	0.608 (0.424–0.872)		0.489 (0.287–0.832)		0.563 (0.195–1.623)	
*p*‐value^c^	0.007		0.008		0.288	

CI, confidence interval; CMAD, cardiac mechanical assist device; CV, cardiovascular; FMQ, US Food and Drug Administration medical query; KM, Kaplan–Meier; NAC, National Amyloidosis Centre; NE, non‐estimable; *TTR*, transthyretin.

^a^ There was no significant interaction between treatment (continuous tafamidis 80 mg or placebo to tafamidis) and NAC stage until the end of study for both all‐cause (*p* for interaction = 0.575) and CV‐related (*p* for interaction = 0.523) mortality.

^b^ Hazard ratio with two‐sided *p*‐value was from a Cox proportional hazards model with treatment and *TTR* genotype (variant and wild‐type) in the model.

^c^ Total annual rate of CV‐related hospitalizations among all patients was calculated as: total CV‐related hospitalizations across all patients divided by total years of study participation across all patients. CV relatedness was based on clinical evaluation and algorithmic assessment of FMQ names and preferred terms.

^d^ Risk ratio and *p*‐value were derived using a Poisson regression model.

### Cardiovascular‐related mortality

**Figure 3:**
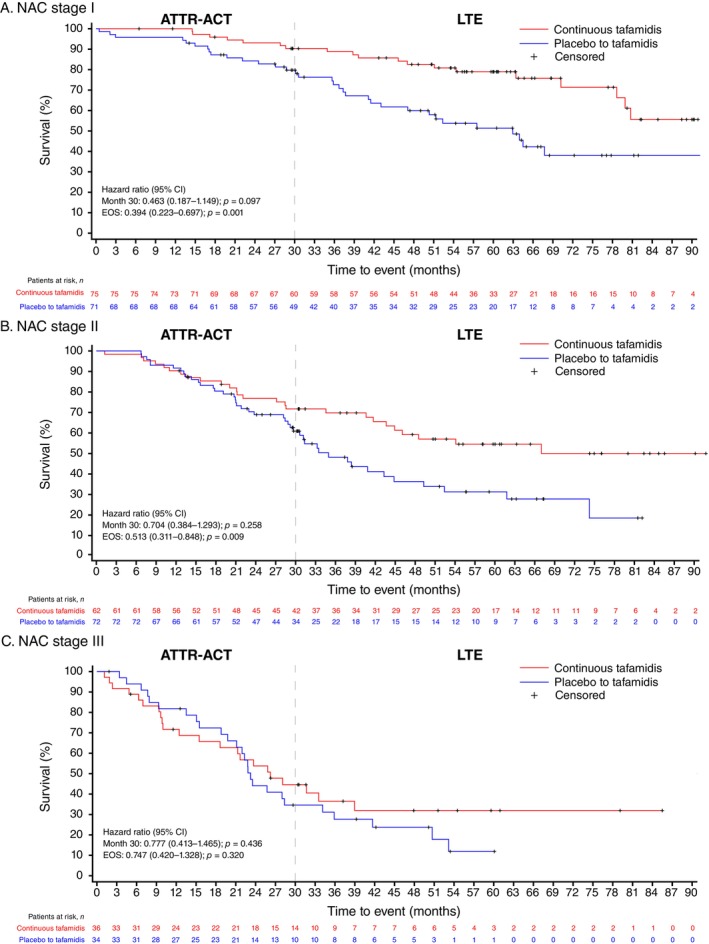
Kaplan–Meier curves of cardiovascular‐related mortality in patients with baseline National Amyloidosis Centre (NAC) stage I–III transthyretin amyloid cardiomyopathy. ATTR‐ACT, Tafamidis in Transthyretin Cardiomyopathy Clinical Trial; CI, confidence interval; EOS, end of study; LTE, long‐term extension study.

Similar patterns were observed for CV‐related mortality. There was no significant interaction between treatment and NAC stage at the EOS (*p* for interaction = 0.523) and at month 30 (*p* for interaction = 0.631). At the end of follow‐up, the proportions with CV‐related mortality in the continuous tafamidis versus placebo to tafamidis groups were 25% versus 46%, 42% versus 58%, and 61% versus 76% for patients at NAC stage I, II, and III, respectively (*Table* *2*). The risk of CV‐related mortality until the EOS was significantly lower in the continuous tafamidis versus placebo to tafamidis groups across NAC stages I (HR [95% CI]: 0.39 [0.22–0.70]; *p* = 0.001) and II (0.51 [0.31–0.85]; *p* = 0.009), with a numerical trend seen in stage III (0.75 [0.42–1.33]; *p* = 0.320) (*Table* *2*). Kaplan–Meier survival curves demonstrated reduction of CV‐related mortality in patients receiving continuous tafamidis treatment, with curves diverging early with tafamidis treatment in those with baseline NAC stage I (*Figure* *3*). Separation of CV‐related survival curves was observed later in patients with NAC stages II and III. Median survival time (95% CI) from CV‐related mortality in patients treated with continuous tafamidis versus placebo to tafamidis across NAC stages was NE (79–NE) versus 63 (43–NE) months for stage I, NE (44–NE) versus 35 (30–45) months for stage II, and 26 (16–39) versus 23 (20–34) months for stage III (*Table* *2*).

### Cardiovascular‐related hospitalizations

The total annual rates (number per year) of CV‐related hospitalizations among all patients were numerically lower in the continuous tafamidis versus placebo to tafamidis groups at all NAC stages: I (0.19 vs. 0.31, relative risk [RR] [95% CI] 0.61 [0.42–0.87]; *p* = 0.007); II (0.14 vs. 0.29, RR 0.49 [0.29–0.83], *p* = 0.008); and III (0.13 vs. 0.23, RR 0.56 [0.20–1.62]; *p* = 0.288) (*Table* *2*).

### Kansas City Cardiomyopathy Questionnaire clinical summary and overall summary scores

The least square mean changes from baseline throughout ATTR‐ACT and the LTE to month 60 in both treatment groups across baseline NAC stages I–III are plotted in online supplementary *Figure* *S1*. While there were no statistically significant differences between treatment groups among patients who were NAC stage III, statistically smaller declines in KCCQ‐OS and ‐CS scores were frequently observed in patients treated continuously with tafamidis versus those who first received placebo at NAC stages I and II. Significant differences were first seen at month 18 for the KCCQ‐OS score in patients at stages I and II and at months 30 and 18 for the KCCQ‐CS score in patients at stages I and II, respectively.

### Sensitivity analyses

ATTR‐ACT was not designed to separate findings by dose. Therefore, we conducted sensitivity analyses on all‐cause mortality, CV‐related mortality, and CV‐related hospitalizations in patients treated continuously with either tafamidis meglumine 80/20 mg (pooled, *n* = 261) throughout the studies versus those treated with placebo in ATTR‐ACT and then tafamidis in the LTE (*n* = 177) across NAC stages I–III (online supplementary *Table* *S2*). There was no significant interaction between treatment and NAC stage until the EOS for all‐cause (*p* for interaction = 0.543) and CV‐related mortality (*p* for interaction = 0.506). Findings from the sensitivity analyses were consistent with the main analyses (online supplementary *Tables* *S3* and *S4*). Additionally, continuous tafamidis 80 mg slightly outperformed the continuous 80/20 mg (pooled) group in terms of mortality risk at NAC stage I, which aligns with a previous study supporting tafamidis 80 mg as the optimal dose.^12^

### Supplementary analysis

To our knowledge, ATTR‐ACT is the only phase 3 trial including patients (*n* = 23) with ATTR‐CM and NT‐proBNP >10 000 ng/L, indicative of very advanced disease and high mortality risk. Using the newly proposed expanded NAC staging system, we performed a supplementary analysis of all‐cause and CV‐related mortality in patients receiving either continuous tafamidis 80 mg or continuous tafamidis 80/20 mg versus placebo to tafamidis across baseline NAC stages I–IV. Of the 350 patients with evaluable data for NAC staging who received tafamidis 80 mg or placebo in ATTR‐ACT, 42%, 36%, 17%, and 5% were NAC stage I, II, III, and IV ATTR‐CM, respectively, at ATTR‐ACT baseline (*Table* *3*, online supplementary *Table* *S5*). Among 438 patients who received tafamidis 80/20 mg (pooled) or placebo in ATTR‐ACT and had evaluable data for NAC staging, 43%, 36%, 16%, and 5% were NAC stage I, II, III, and IV at ATTR‐ACT baseline. There was no significant interaction between treatment and NAC stage until the EOS for both all‐cause and CV‐related mortality (*p* for interaction ≥0.3). Overall, findings from the supplementary analysis were similar to the main analysis showing reduced all‐cause and CV‐related mortality with continuous tafamidis treatment versus placebo to tafamidis in patients at NAC stages I–II and a numerically favourable trend in those at higher NAC stages III–IV, with survival curves diverging early in patients at baseline NAC stage I (*Table* *3*, online supplementary *Figures* *S2* and *S3*). Significant risk reductions in both all‐cause and CV‐related mortality were observed in the continuous tafamidis 80 mg or pooled tafamidis 80/20 mg group versus the placebo to tafamidis group at baseline NAC stages I and II (*Table* *3*). Despite the small sample size of patients at baseline NAC stages III and IV, continuous treatment with tafamidis 80 mg or 80/20 mg (pooled) showed a numerical decrease in all‐cause and CV‐related mortality, with survival curves diverging later during the LTE (*Table* *3*, online supplementary *Figures* *S2* and *S3*).

**Table 3:** All‐cause and cardiovascular‐related mortality in the Tafamidis in Transthyretin Cardiomyopathy Clinical Trial and the long‐term extension study across expanded National Amyloidosis Centre stages I–IV until the end of study

	NAC stage I			NAC stage II			NAC stage III			NAC stage IV		
	Placebo to tafamidis^a^ (*n* = 71)	Continuous tafamidis 80 mg^a^ (*n* = 75)	Continuous tafamidis 80/20 mg (pooled)^a^ (*n* = 118)	Placebo to tafamidis^a^ (*n* = 69)	Continuous tafamidis 80 mg^a^ (*n* = 58)	Continuous tafamidis 80/20 mg (pooled)^a^ (*n* = 87)	Placebo to tafamidis^a^ (*n* = 29)	Continuous tafamidis 80 mg^a^ (*n* = 32)	Continuous tafamidis 80/20 mg (pooled)^a^ (*n* = 41)	Placebo to tafamidis^a^ (*n* = 8)	Continuous tafamidis 80 mg^a^ (*n* = 8)	Continuous tafamidis 80/20 mg (pooled)^a^ (*n* = 15)
All‐cause mortality, *n* (%)^b^, ^c^	43 (60.6)	27 (36.0)	53 (44.9)	50 (72.5)	31 (53.4)	47 (54.0)	25 (86.2)	23 (71.9)	30 (73.2)	8 (100.0)	5 (62.5)	12 (80.0)
Death	40 (56.3)	23 (30.7)	48 (40.7)	48 (69.6)	28 (48.3)	42 (48.3)	24 (82.8)	21 (65.6)	28 (68.3)	8 (100.0)	5 (62.5)	12 (80.0)
Heart transplant	3 (4.2)	4 (5.3)	5 (4.2)	2 (2.9)	3 (5.2)	5 (5.7)	1 (3.4)	0	0	0	0	0
CMAD	0	0	0	0	0	0	0	2 (6.3)	2 (4.9)	0	0	0
KM estimate of time to event, months, median (95% CI)	51.3 (37.7–66.7)	80.8 (70.2–NE)	70.2 (66.2–80.8)	32.5 (26.2–38.4)	54.1 (36.7–NE)	52.3 (40.6–NE)	23.2 (18.8–34.1)	24.8 (10.0–31.7)	23.7 (13.0–33.5)	8.7 (1.8–30.6)	34.8 (1.2–NE)	20.7 (5.6–41.1)
Hazard ratio for continuous tafamidis vs. placebo to tafamidis (95% CI)^d^	–	0.422 (0.260–0.685)	0.582 (0.388–0.872)	–	0.511 (0.323–0.806)	0.499 (0.331–0.752)	–	0.878 (0.495–1.558)	0.850 (0.498–1.452)	–	0.709 (0.199–2.523)	1.079 (0.376–3.095)
*p*‐value^d^	–	0.001	0.009	–	0.004	0.001	–	0.658	0.552	–	0.595	0.887
CV‐related mortality, *n* (%)^b^, ^c^	33 (46.5)	19 (25.3)	38 (32.2)	39 (56.5)	23 (39.7)	34 (39.1)	22 (75.9)	20 (62.5)	26 (63.4)	7 (87.5)	5 (62.5)	11 (73.3)
Death	30 (42.3)	15 (20.0)	33 (28.0)	37 (53.6)	20 (34.5)	29 (33.3)	21 (72.4)	18 (56.3)	24 (58.5)	7 (87.5)	5 (62.5)	11 (73.3)
Heart transplant	3 (4.2)	4 (5.3)	5 (4.2)	2 (2.9)	3 (5.2)	5 (5.7)	1 (3.4)	0	0	0	0	0
CMAD	0 (0.0)	0 (0.0)	0 (0.0)	0	0	0	0 (0.0)	2 (6.3)	2 (4.9)	0	0	0
KM estimate of time to event, months, median (95% CI)	62.9 (43.0–NE)	NE (78.7–NE)	79.9 (69.6–NE)	37.8 (29.7–49.3)	NE (44.9–NE)	92.3 (48.7–NE)	23.5 (19.8–34.1)	26.2 (12.5–39.0)	26.2 (18.6–50.9)	9.3 (7.6–30.6)	34.8 (1.2–NE)	21.1 (6.2–48.5)
Hazard ratio for continuous tafamidis vs. placebo to tafamidis (95% CI)^d^	–	0.390 (0.221–0.688)	0.543 (0.339–0.868)	–	0.505 (0.299–0.852)	0.472 (0.294–0.758)	–	0.826 (0.447–1.525)	0.812 (0.458–1.440)	–	0.794 (0.210–2.997)	1.174 (0.374–3.683)
*p*‐value^d^	–	0.001	0.011	–	0.011	0.002	–	0.541	0.476	–	0.733	0.784

CI, confidence interval; CMAD, cardiac mechanical assist device; CV, cardiovascular; KM, Kaplan–Meier; LTE, long‐term extension study; NAC, National Amyloidosis Centre; *TTR*, transthyretin.

^a^ Following a protocol amendment, all patients in the LTE transitioned to tafamidis free acid 61 mg (bioequivalent to tafamidis meglumine 80 mg).

^b^ There was no significant interaction between treatment (continuous tafamidis 80 mg or placebo to tafamidis) and NAC stage until the end of study for both all‐cause (*p* for interaction = 0.306) and CV‐related (*p* for interaction = 0.449) mortality.

^c^ There was no significant interaction between treatment (continuous tafamidis 80/20 mg [pooled] or placebo to tafamidis) and NAC stage until the end of study for both all‐cause (*p* for interaction = 0.604) and CV‐related (*p* for interaction = 0.630) mortality.

^d^ Hazard ratio with two‐sided *p*‐value was from a Cox proportional hazards model with treatment and *TTR* genotype (variant and wild‐type) in the model.

## Discussion

Findings from this post‐hoc analysis are consistent with previous studies supporting the benefit of tafamidis in patients with ATTR‐CM. Continuous tafamidis treatment was associated with reductions in all‐cause and CV‐related mortality, CV‐related hospitalizations, symptom worsening, and lesser decline in health‐related quality of life in patients at NAC stages I and II, with a numerical trend observed in NAC stage III. Treatment benefits were seen earlier and were of a greater magnitude in patients with an earlier disease stage at baseline.^10^, ^11^, ^12^, ^14^, ^15^

Disease‐modifying therapies for patients with ATTR‐CM might have a different impact at various disease stages. However, the effects of disease‐modifying treatment across the spectrum of ATTR‐CM severity are often poorly evaluated in clinical trial data, as patients with advanced disease are often excluded.^6^, ^8^, ^16^, ^17^, ^18^ The HELIOS‐B trial did not include patients with NYHA class III (alongside NAC stage III) or IV, or an eGFR <30 ml/min/1.73 m^2^.^17^ The ATTRibute‐CM trial excluded patients with NT‐proBNP ≥8500 ng/L or an eGFR <15 ml/min/1.73 m^2^.^18^ To our knowledge, ATTR‐ACT is the only phase 3 trial that included patients with severe ATTR‐CM and therefore is the only trial able to provide information on treatment effects in a wide spectrum of disease severity.^10^

In the current analysis, continuous tafamidis meglumine 80 mg or free acid 61 mg versus placebo in ATTR‐ACT then tafamidis in the LTE was associated with a significantly reduced risk of all‐cause mortality among patients with baseline NAC stages I (57%) and II (49%) at the end of the study. The benefit of tafamidis was seen earlier (approximately as early as 18 months in NAC stage I and 24 months in stage II) and was larger over the duration of follow‐up in patients with lower staging. Comparable risk reductions in CV‐related mortality were also observed in patients with baseline NAC stages I (61%) and II (49%). Similarly, continuous tafamidis treatment showed lower risk of CV‐related hospitalizations in patients with baseline NAC stages I (33%) and II (43%). Positive results associated with continuous tafamidis versus placebo to tafamidis treatment reached statistical significance only in patients with lower baseline NAC stages I and II. However, a numerical trend suggested potential benefit from continuous tafamidis treatment in patients with advanced ATTR‐CM (NAC stages III or IV).

The approved dose of tafamidis has been shown to have the greatest clinical benefit in patients who start treatment at an earlier disease stage.^12^, ^19^, ^20^, ^21^, ^22^ Despite this, previous studies have also demonstrated the value of tafamidis treatment in patients with ATTR‐CM and severe HF symptoms (i.e. NYHA class III).^19^, ^23^, ^24^ Supporting these previous findings, results from our study showed significantly larger reductions in all‐cause and CV‐related mortality and lesser declines in KCCQ‐OS and ‐CS scores in patients who received continuous tafamidis versus placebo to tafamidis treatment and were NAC stages I and II at baseline. This highlights the importance of early diagnosis and administration of disease‐modifying therapy for patients with ATTR‐CM. Although not statistically significant in patients at baseline NAC stage III (or in the new stage IV), numerical trends for reduced all‐cause and CV‐related mortality risk, a lower risk of CV‐related hospitalizations, and a lesser decline in KCCQ‐OS and ‐CS scores were observed in the continuous tafamidis versus placebo to tafamidis groups. Findings from this study provide additional support on the treatment benefits of tafamidis for patients with early and advanced ATTR‐CM.

### Limitations

This post‐hoc analysis was not pre‐specified, and neither ATTR‐ACT nor the LTE was statistically powered to address the impact of tafamidis treatment by NAC stage. Also, the number of patients with ATTR‐CM across baseline NAC stages was limited, particularly for NAC stages III and IV; therefore, the results are exploratory and need to be interpreted with caution. ATTR‐ACT was not designed to separate findings by tafamidis dose. Thus, we included the pooled tafamidis meglumine 80 or 20 mg group for the sensitivity analyses. Using the whole dataset increased the sample size but results were comparable. Further, our findings add evidence that continuous tafamidis 80 mg is associated with better outcomes than 20 mg over a longer treatment period. All patients received tafamidis in the LTE and so the observed differences in outcomes might be less if compared with a true placebo arm.

## Conclusions

Continuous tafamidis treatment reduced all‐cause mortality, CV‐related mortality, and CV‐related hospitalizations, and minimized the worsening of symptoms and decline in quality of life among patients with ATTR‐CM in NAC stages I and II. These reductions were largest and occurred earliest in patients at NAC stage I. Among patients in NAC stages III and IV, there was a non‐significant numerical trend observed towards better outcomes in those receiving continuous tafamidis versus delayed tafamidis treatment. Findings from this study further emphasize the importance of early diagnosis and initiation of disease‐modifying therapy in patients with ATTR‐CM.

## Funding

This study was sponsored by Pfizer.

**Conflict of interest**: T.D. has received consulting fees from Alnylam, GlaxoSmithKline, Pfizer, and Prothena; honoraria from Alnylam, Pfizer, and Prothena; research grants from GlaxoSmithKline and Pfizer; and clinical trial support from Alnylam, Ionis Pharmaceuticals, and Pfizer. R.W. is an employee of Pfizer and holds shares/share options. M.S.M. has received research grants and personal fees from Alnylam, Eidos Therapeutics, Attralus, Intellia Therapeutics, Ionis Pharmaceuticals, and Pfizer; and personal fees from Akcea Therapeutics, AstraZeneca, Attralus, Intellia Therapeutics, and Novo Nordisk. J.D.G. has received consultancy fees from Alexion, Alnylam, AstraZeneca, Attralus, BridgeBio/Eidos Therapeutics, Intellia Therapeutics, Ionis Pharmaceuticals, Lycia Therapeutics, and Pfizer; and received research grants from Alnylam, AstraZeneca, and BridgeBio. M.F. reports being on consultancy/advisory boards for Alexion/Caelum Biosciences, Alnylam, AstraZeneca, Attralus, BridgeBio/Eidos Therapeutics, Cardior, Intellia Therapeutics, Ionis Pharmaceuticals, Lexeo Therapeutics, Janssen, Novo Nordisk, Pfizer, and Prothena; receiving research grants from Alnylam, AstraZeneca, BridgeBio, and Pfizer; and receiving salary from a British Heart Foundation Intermediate Fellowship.

## Supporting information


**Figure S1.** Change from baseline in Kansas city cardiomyopathy questionnaire‐overall summary and clinical summary (KCCQ‐OS/CS) and overall summary (OS) scores across National Amyloidosis Centre (NAC) stages I–III.

**Figure S2.** Kaplan–Meier curves of all‐cause mortality in patients with baseline National Amyloidosis Centre (NAC) stages I–IV approved treatment for patients with transthyretin amyloid cardiomyopathy (ATTR‐CM).

**Figure S3.** Kaplan–Meier curves of cardiovascular‐related mortality in patients with baseline National Amyloidosis Centre (NAC) stages I–IV approved treatment for patients with transthyretin amyloid cardiomyopathy (ATTR‐CM).

**Table S1.** US Food and Drug Administration medical query (FMQ) names and cardiovascular (CV)‐related preferred terms.

**Table S2.** Sensitivity analysis baseline characteristics of patients across National Amyloidosis Centre (NAC) stages I–III.

**Table S3.** Sensitivity analysis on all‐cause and cardiovascular (CV)‐related mortality in tafamidis in transthyretin cardiomyopathy clinical trial (ATTR‐ACT) and the long‐term extension study (LTE) across baseline National Amyloidosis Centre (NAC) stages I–III.

**Table S4.** Sensitivity analysis on cardiovascular (CV)‐related hospitalizations across baseline National Amyloidosis Centre (NAC) stages I–III.

**Table S5.** Baseline patient characteristics across expanded National Amyloidosis Centre (NAC) stages I–IV.

**Data S1.** Supporting Information.

## Data Availability

Upon request, and subject to review, Pfizer will provide the data that support the findings of this study. Subject to certain criteria, conditions, and exceptions, Pfizer may also provide access to the related individual de‐identified participant data. See https://www.pfizer.com/science/clinical‐trials/trial‐data‐and‐results for more information.
