# Supplementary material for: Long‐term efficacy of tafamidis in patients with transthyretin amyloid cardiomyopathy by National Amyloidosis Centre stage
Source: Eur J Heart Fail. 2025 Jun 9;27(12):2998–3009. doi: 10.1002/ejhf.3696 (PMC12803551; doi:10.1002/ejhf.3696)
Supplement: Supplementary file 1 — Figure S1. Change from baseline in Kansas city cardiomyopathy questionnaire‐overall summary and clinical summary (KCCQ‐OS/CS) and overall summary (OS) scores across National Amyloidosis Centre (NAC) stages I–III. [file EJHF-27-2998-s007.pdf]

Figure S1 Change from baseline in KCCQ-CS and -OS scores across NAC stages I–III

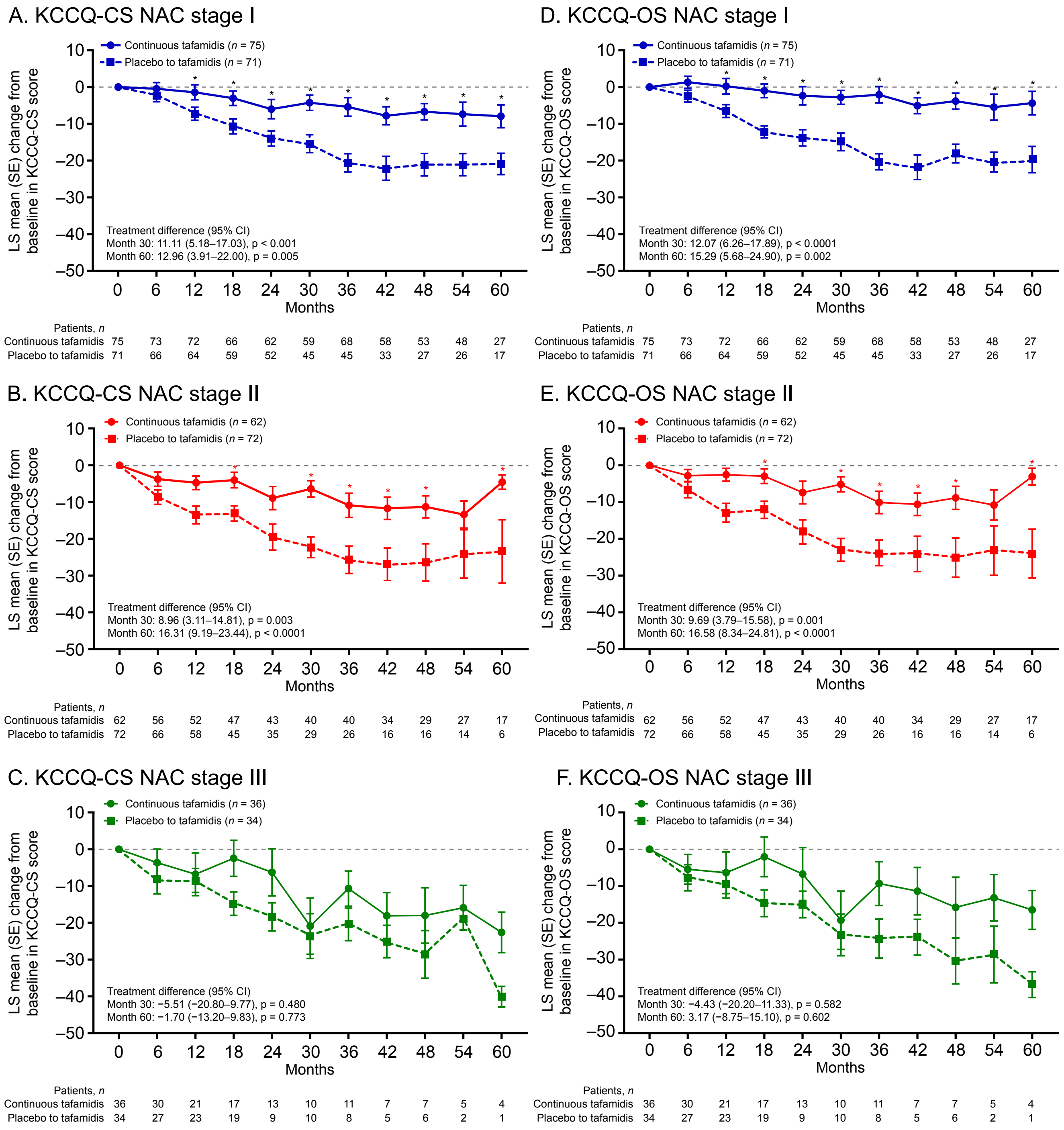

The analysis was based on a mixed model for repeated measures with an unstructured covariance matrix. Treatment, visit, *TTR* genotype, and visit-by-treatment interaction were treated as fixed effects. CS was calculated as the mean of physical limitation, symptom frequency, and symptom burden scores. OS was calculated as the mean of physical limitation, symptom frequency, symptom burden, quality of life, and social limitation scores.

*$p \leq 0.05$ for the LS mean difference from the placebo to tafamidis treatment group.

CS, clinical summary; KCCQ, Kansas City Cardiomyopathy Questionnaire; LS, least square; NAC, National Amyloidosis Centre; OS, overall summary; SE, standard error; *TTR*, transthyretin.
