# Supplementary material for: Long‐term efficacy of tafamidis in patients with transthyretin amyloid cardiomyopathy by National Amyloidosis Centre stage
Source: Eur J Heart Fail. 2025 Jun 9;27(12):2998–3009. doi: 10.1002/ejhf.3696 (PMC12803551; doi:10.1002/ejhf.3696)
Supplement: Supplementary file 3 — Figure S3. Kaplan–Meier curves of cardiovascular‐related mortality in patients with baseline National Amyloidosis Centre (NAC) stages I–IV approved treatment for patients with transthyretin amyloid cardiomyopathy (ATTR‐CM). [file EJHF-27-2998-s004.pdf]

Figure S3 Kaplan-Meier curves of cardiovascular-related mortality in patients with baseline NAC stages I–IV ATTR-CM

A. NAC stage I

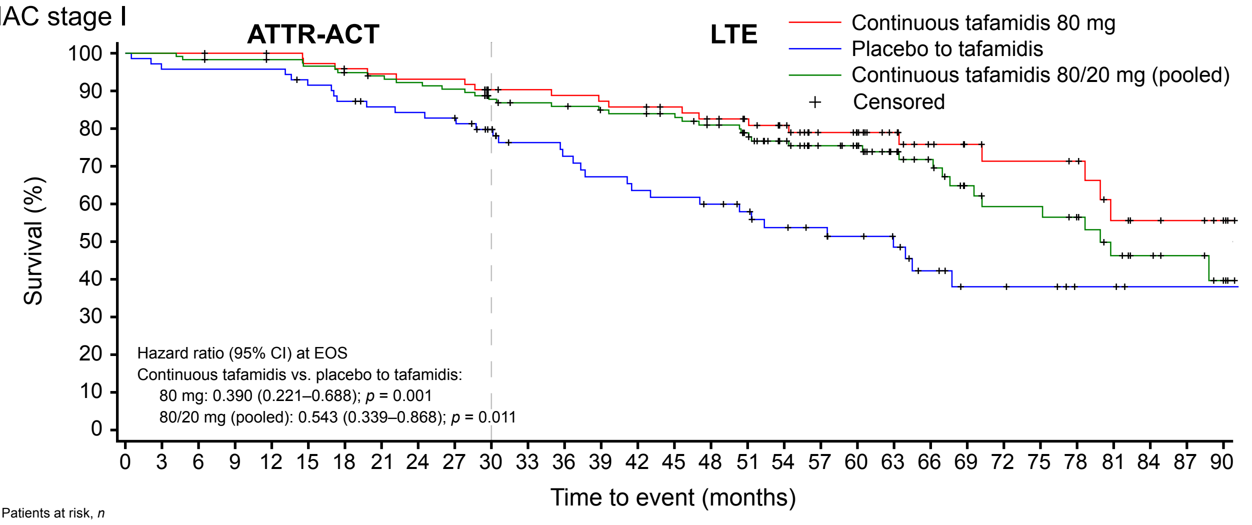

Patients at risk, *n*																															
Continuous tafamidis 80 mg	75	75	75	74	73	71	69	68	67	67	60	59	58	57	56	54	51	48	44	36	33	27	21	18	16	16	15	10	8	7	4
Continuous tafamidis 80/20 mg (pooled)	118	118	116	115	114	112	109	107	105	103	94	91	90	87	86	84	79	73	63	54	48	39	32	24	21	21	18	13	10	8	4
Placebo to tafamidis	71	68	68	68	68	64	61	58	57	56	49	42	40	37	35	34	32	29	25	23	20	17	12	8	8	7	4	4	2	2	2

B. NAC stage II

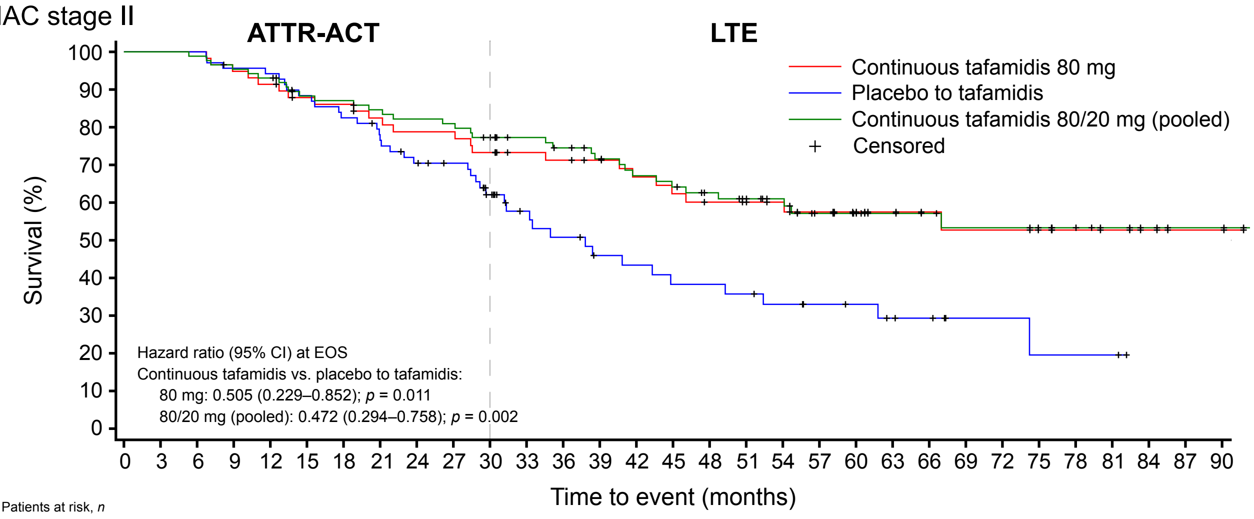

Patients at risk, *n*																															
Continuous tafamidis 80 mg	58	58	58	55	53	49	48	45	43	43	40	36	35	33	30	28	26	25	23	20	17	14	12	11	11	9	7	6	4	2	2
Continuous tafamidis 80/20 mg (pooled)	87	87	86	82	80	73	72	69	67	66	62	56	53	49	45	43	39	36	32	26	22	18	16	14	14	12	10	7	5	3	3
Placebo to tafamidis	69	69	69	66	65	60	56	51	46	43	33	25	22	18	17	15	15	14	12	10	9	7	6	3	3	2	2	2	0	0	0

C. NAC stage III

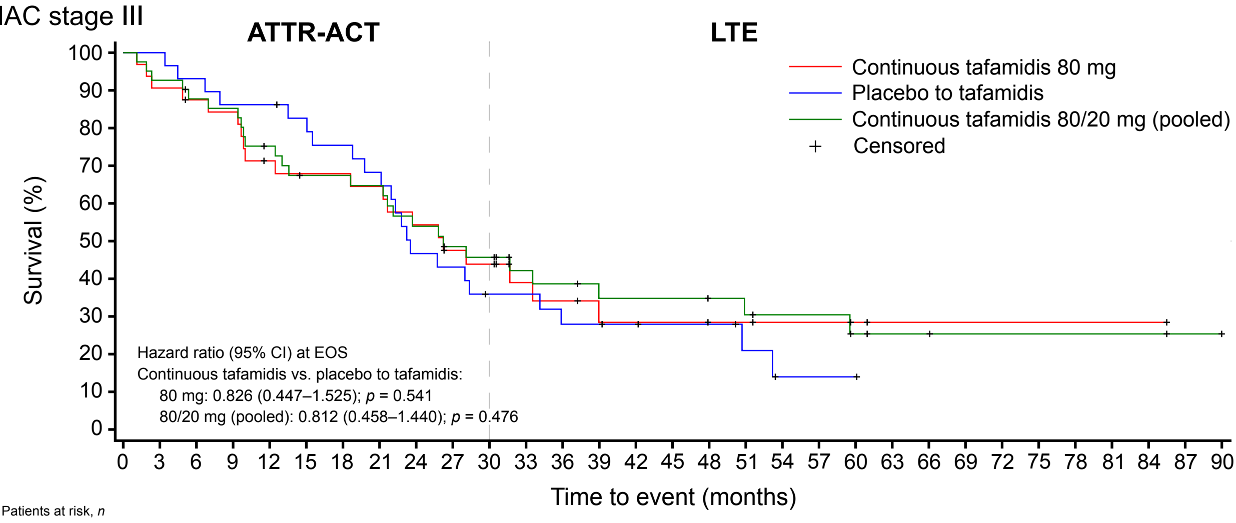

Patients at risk, *n*																															
Continuous tafamidis 80 mg	32	29	27	26	21	20	20	19	16	13	12	8	7	5	5	5	4	4	3	3	2	1	1	1	1	1	1	1	1	0	0
Continuous tafamidis 80/20 mg (pooled)	41	38	35	34	29	25	25	24	20	17	16	12	11	9	9	9	8	7	6	6	4	3	3	2	2	2	2	2	2	1	0
Placebo to tafamidis	29	29	27	25	25	23	21	19	13	12	9	9	7	7	6	5	5	3	1	1	1	0	0	0	0	0	0	0	0	0	0

D. NAC stage IV

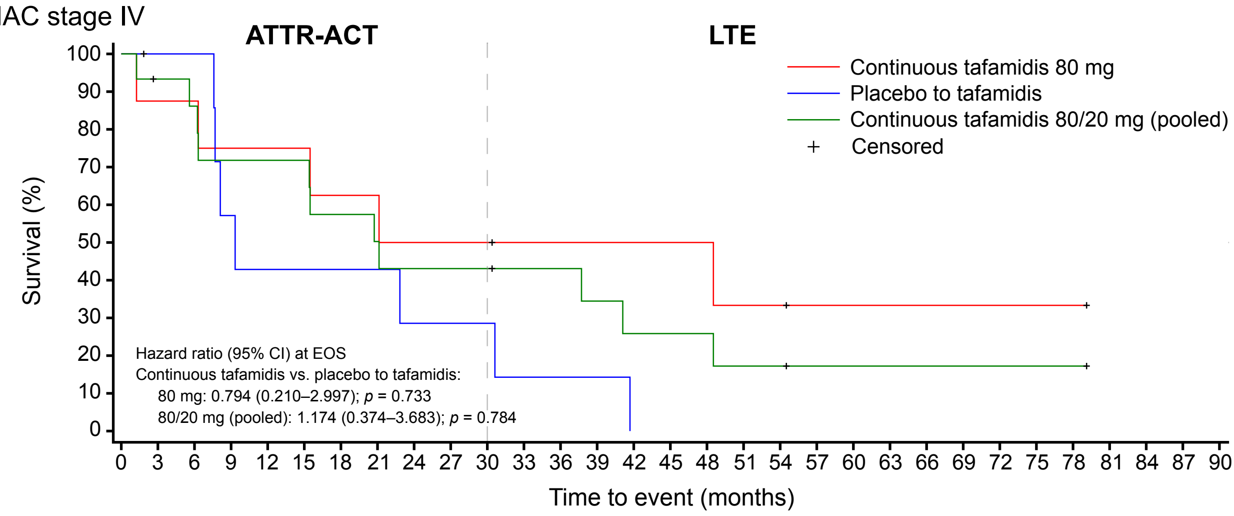

Patients at risk, *n*																															
Continuous tafamidis 80 mg	8	7	7	6	6	6	5	5	4	4	4	3	3	3	3	3	3	2	2	1	1	1	1	1	1	1	1	0	0	0	0
Continuous tafamidis 80/20 mg (pooled)	15	13	12	10	10	10	8	7	6	6	6	5	5	4	3	3	3	2	2	1	1	1	1	1	1	1	1	0	0	0	0
Placebo to tafamidis	8	7	7	4	3	3	3	3	2	2	2	1	1	1	0	0	0	0	0	0	0	0	0	0	0	0	0	0	0	0	0

ATTR-ACT, Tafamidis in Transthyretin Cardiomyopathy Clinical Trial; ATTR-CM, transthyretin amyloid cardiomyopathy; CI, confidence interval; EOS, end of study; LTE, long-term extension study; NAC, National Amlyoidosis Centre
