# Supplementary material for: Long‐term efficacy of tafamidis in patients with transthyretin amyloid cardiomyopathy by National Amyloidosis Centre stage
Source: Eur J Heart Fail. 2025 Jun 9;27(12):2998–3009. doi: 10.1002/ejhf.3696 (PMC12803551; doi:10.1002/ejhf.3696)
Supplement: Supplementary file 4 — Table S1. US Food and Drug Administration medical query (FMQ) names and cardiovascular (CV)‐related preferred terms. [file EJHF-27-2998-s001.docx]

| **Table S1 FMQ names and CV-related preferred terms** | |
| --- | --- |
| **FMQ names** | Heart failure, peripheral oedema; arrhythmia, cardiac conduction disturbance, palpitations, tachycardia; myocardial infarction, myocardial ischaemia; stroke and transient ischaemic attack; thrombosis, venous thrombosis, thrombosis arterial; syncope, hypotension, system hypertension |
| **Additional CV-related preferred terms** | Aortic valve stenosis, aortic stenosis, aortic dissection, amyloidosis senile, cardiac amyloidosis, cardiac arrest, cardioversion, cardiovascular insufficiency, chronotropic incompetence, chest pain, circulatory collapse, complications from heart transplant, endocarditis, disease progression, ATTR disease progression, familial amyloidosis, pulmonary hypertension, peripheral venous disease, peripheral arterial occlusive disease, pericarditis infective, pericardial effusion, pericarditis, peripheral ischaemia, primary amyloidosis, pulseless electrical activity, sudden cardiac death, sudden arrhythmic death, sudden death, tricuspid valve incompetence |
| ATTR, transthyretin amyloidosis; CV, cardiovascular; FDA, US Food and Drug Administration; FMQ, FDA medical query | |
