# Supplementary material for: Long‐term efficacy of tafamidis in patients with transthyretin amyloid cardiomyopathy by National Amyloidosis Centre stage
Source: Eur J Heart Fail. 2025 Jun 9;27(12):2998–3009. doi: 10.1002/ejhf.3696 (PMC12803551; doi:10.1002/ejhf.3696)
Supplement: Supplementary file 5 — Table S2. Sensitivity analysis baseline characteristics of patients across National Amyloidosis Centre (NAC) stages I–III. [file EJHF-27-2998-s002.docx]

| **Table S2 Sensitivity analysis baseline characteristics of patients across NAC stages I–III** | | | | | | |
| --- | --- | --- | --- | --- | --- | --- |
|  | **NAC stage I** | | **NAC stage II** | | **NAC stage III** | |
|  | **Placebo to tafamidis^a^ (*n* = 71)** | **Continuous tafamidis 80/20 mg (pooled)^a^** (***n* = 118)** | **Placebo to tafamidis^a^ (*n* = 72)** | **Continuous tafamidis 80/20 mg (pooled)^a^ (*n* = 93)** | **Placebo to tafamidis^a^ (*n* = 34)** | **Continuous tafamidis 80/20 mg (pooled)^a^ (*n* = 50)** |
| **Age, mean (SD), years** | 72.1 (7.4) | 73.4 (7.2) | 74.6 (5.7) | 74.5 (7.1) | 77.0 (6.0) | 77.4 (7.0) |
| **Sex, n (%)** |  |  |  |  |  |  |
| Male | 62 (87.3) | 111 (94.1) | 66 (91.7) | 85 (91.4) | 29 (85.3) | 42 (84.0) |
| Female | 9 (12.7) | 7 (5.9) | 6 (8.3) | 8 (8.6) | 5 (14.7) | 8 (16.0) |
| **Race, n (%)** |  |  |  |  |  |  |
| White | 61 (85.9) | 101 (85.6) | 61 (84.7) | 77 (82.8) | 24 (70.6) | 30 (60.0) |
| Black | 9 (12.7) | 11 (9.3) | 9 (12.5) | 10 (10.8) | 8 (23.5) | 16 (32.0) |
| Asian | 1 (1.4) | 5 (4.2) | 2 (2.8) | 6 (6.5) | 2 (5.9) | 2 (4.0) |
| American Indian or Alaska Native | 0 | 1 (0.8) | 0 | 0 | 0 | 2 (4.0) |
| ***TTR* genotype, n (%)** |  |  |  |  |  |  |
| Wild type | 52 (73.2) | 97 (82.2) | 57 (79.2) | 71 (76.3) | 25 (73.5) | 30 (60.0) |
| Variant | 19 (26.8) | 21 (17.8) | 15 (20.8) | 22 (23.7) | 9 (26.5) | 20 (40.0) |
| **NT-proBNP,** **mean (SD), ng/L** | 1837.3 (665.5) | 1733.1 (675.2) | 4533.9 (2717.8) | 4972.0 (3089.9) | 6580.5 (3583.1) | 7215.3 (4102.5) |
| **mBMI, mean (SD)^b^** | 1094 (197.7) | 1085 (175.2) | 1019 (185.6) | 1019 (163.5) | 1110 (190.1) | 1063 (176.3) |
| **eGFR, mean (SD),** **ml/min/1.73m^2c^** | 66.0 (13.4) | 68.0 (13.8) | 54.8 (14.0) | 56.1 (12.2) | 35.3 (6.3) | 34.4 (6.5) |
| **Troponin I, mean (SD), ng/ml** | 0.1 (0.2) | 0.2 (0.2) | 0.2 (0.2)^d^ | 0.2 (0.6) | 0.2 (0.2) | 0.7 (1.8) |
| **NYHA class, n (%)** |  |  |  |  |  |  |
| I | 6 (8.5) | 14 (11.9) | 6 (8.3) | 7 (7.5) | 1 (2.9) | 2 (4.0) |
| II | 48 (67.6) | 82 (69.5) | 39 (54.2) | 58 (62.4) | 14 (41.2) | 22 (44.0) |
| III | 17 (23.9) | 22 (18.6) | 27 (37.5) | 28 (30.1) | 19 (55.9) | 26 (52.0) |
| **Follow-up duration, median (95% CI), months** | 64.2 (57.5–77.8) | 63.3 (60.1–68.8) | 59.1 (51.6–81.5) | 60.4 (56.6–74.3) | 53.4 (50.2–NE) | 59.6 (47.9–79.1) |
| ^a^Following a protocol amendment, all patients in the LTE transitioned to tafamidis free acid 61 mg (bioequivalent to tafamidis meglumine 80 mg).  ^b^mBMI was calculated as the serum albumin level (g/L) multiplied by the BMI (weight in kg/square of the height in metres).  ^c^eGFR was estimated using the Chronic Kidney Disease Epidemiology Collaboration (CKD-EPI) equation.  ^d^*n* = 71.  ^e^Calculated using the Kaplan–Meier method.  BMI, body mass index; CI, confidence interval; eGFR, estimated glomerular filtration rate; LTE, long-term extension study; mBMI, modified BMI; NAC, National Amyloidosis Centre; NE, non-estimable; NT-proBNP, N-terminal pro-B-type natriuretic peptide; SD, standard deviation; *TTR*, transthyretin. | | | | | | |
