# Supplementary material for: Long‐term efficacy of tafamidis in patients with transthyretin amyloid cardiomyopathy by National Amyloidosis Centre stage
Source: Eur J Heart Fail. 2025 Jun 9;27(12):2998–3009. doi: 10.1002/ejhf.3696 (PMC12803551; doi:10.1002/ejhf.3696)
Supplement: Supplementary file 6 — Table S3. Sensitivity analysis on all‐cause and cardiovascular (CV)‐related mortality in tafamidis in transthyretin cardiomyopathy clinical trial (ATTR‐ACT) and the long‐term extension study (LTE) across baseline National Amyloidosis Centre (NAC) stages I–III. [file EJHF-27-2998-s008.docx]

| **Table S3 Sensitivity analysis on all-cause and CV-related mortality in ATTR-ACT and the LTE across baseline NAC stages I–III** | | | | | | |
| --- | --- | --- | --- | --- | --- | --- |
|  | **NAC stage I** | | **NAC stage II** | | **NAC stage III** | |
|  | **Placebo to tafamidis^a^** (***n* = 71)** | **Continuous tafamidis 80/20 mg (pooled)^a^** (***n* = 118)** | **Placebo to tafamidis^a^** (***n* = 72)** | **Continuous tafamidis 80/20 mg (pooled)^a^** (***n* = 93)** | **Placebo to tafamidis^a^** (***n* = 34)** | **Continuous tafamidis 80/20 mg (pooled)^a^** (***n* = 50)** |
| **All-cause mortality, n (%)^b^** | 43 (60.6) | 53 (44.9) | 53 (73.6) | 52 (55.9) | 30 (88.2) | 37 (74.0) |
| Death | 40 (56.3) | 48 (40.7) | 51 (70.8) | 47 (50.5) | 29 (85.3) | 35 (70.0) |
| Heart transplant | 3 (4.2) | 5 (4.2) | 2 (2.8) | 5 (5.4) | 1 (2.9) | 0 |
| CMAD | 0 | 0 | 0 | 0 | 0 | 2 (4.0) |
| KM estimate of time to event, median (95% CI), months | 51.3 (37.7–66.7) | 70.2 (66.2–80.8) | 31.3 (26.2–37.8) | 48.5 (40.6–85.6) | 22.8 (15.5–28.4) | 21.9 (13.0–31.7) |
| Hazard ratio for continuous tafamidis vs. placebo to tafamidis (95% CI)^c^ | 0.576 (0.384–0.865) | | 0.492 (0.330–0.734) | | 0.805 (0.495–1.308) | |
| *p*-value^c^ | 0.008 | | 0.001 | | 0.381 | |
| **CV-related mortality, n (%)^b^** | 33 (46.5) | 38 (32.2) | 42 (58.3) | 39 (41.9) | 26 (76.5) | 32 (64.0) |
| Death | 30 (42.3) | 33 (28.0) | 40 (55.6) | 34 (36.6) | 25 (73.5) | 30 (60.0) |
| Heart transplant | 3 (4.2) | 5 (4.2) | 2 (2.8) | 5 (5.4) | 1 (2.9) | 0 |
| CMAD | 0 | 0 | 0 | 0 | 0 | 2 (4.0) |
| KM estimate of time to event, median (95% CI), months | 62.9 (43.0–NE) | 79.9 (69.6–NE) | 35.0 (29.7–44.8) | 92.3 (46.1–NE) | 23.2 (19.8–34.1) | 25.8 (15.5–39.0) |
| Hazard ratio for continuous tafamidis vs. placebo to tafamidis (95% CI)^c^ | 0.541 (0.338–0.865) | | 0.478 (0.304–0.751) | | 0.795 (0.472–1.340) | |
| *p*-value^c^ | 0.010 | | 0.001 | | 0.390 | |
| ^a^Following a protocol amendment, all patients in the LTE transitioned to tafamidis free acid 61 mg (bioequivalent to tafamidis meglumine 80 mg).  ^b^There was no significant interaction between treatment (continuous tafamidis 80/20 mg [pooled] or placebo to tafamidis) and NAC stage until the end of study for both all-cause (*p* for interaction = 0.543) and CV-related (*p* for interaction = 0.506) mortality.  ^c^Hazard ratio with two-sided *p*-value was from a Cox proportional hazards model with treatment and *TTR* genotype (variant and wild-type) in the model.  ATTR-ACT, Tafamidis in Transthyretin Cardiomyopathy Clinical Trial; CI, confidence interval; CMAD, cardiac mechanical assist device; CV, cardiovascular; KM, Kaplan–Meier; LTE, long-term extension study; NAC, National Amyloidosis Centre; *TTR*, transthyretin. | | | | | | |
