# Supplementary material for: Long‐term efficacy of tafamidis in patients with transthyretin amyloid cardiomyopathy by National Amyloidosis Centre stage
Source: Eur J Heart Fail. 2025 Jun 9;27(12):2998–3009. doi: 10.1002/ejhf.3696 (PMC12803551; doi:10.1002/ejhf.3696)
Supplement: Supplementary file 7 — Table S4. Sensitivity analysis on cardiovascular (CV)‐related hospitalizations across baseline National Amyloidosis Centre (NAC) stages I–III. [file EJHF-27-2998-s006.docx]

| **Table S4. Sensitivity analysis on CV-related hospitalisations across baseline NAC stages I–III** | | | | | | |
| --- | --- | --- | --- | --- | --- | --- |
|  | **NAC stage I** | | **NAC stage II** | | **NAC stage III** | |
|  | **Placebo to tafamidis^a^** (***n* = 71)** | **Continuous tafamidis 80/20 mg (pooled)^a^** (***n* = 118)** | **Placebo to tafamidis^a^** (***n* = 72)** | **Continuous tafamidis 80/20 mg (pooled)^a^** (***n* = 93)** | **Placebo to tafamidis^a^** (***n* = 34)** | **Continuous tafamidis 80/20 mg (pooled)^a^**  (***n* = 50)** |
| **CV-related hospitalisations** |  |  |  |  |  |  |
| Patients with ≥1 CV-related hospitalisations, n (%) | 23 (32.4) | 45 (38.1) | 15 (20.8) | 28 (30.1) | 5 (14.7) | 11 (22.0) |
| Total annual rate of CV-related hospitalisations among all patients, n^b^ | 0.31 | 0.20 | 0.29 | 0.14 | 0.23 | 0.21 |
| Risk ratio for continuous tafamidis vs. placebo to tafamidis (95% CI)^c^ | 0.655 (0.475–0.904) | | 0.492 (0.304–0.798) | | 0.881 (0.373–2.078) | |
| *p*-value^c^ | 0.010 | | 0.004 | | 0.772 | |
| ^a^Following a protocol amendment, all patients in the LTE transitioned to tafamidis free acid 61 mg (bioequivalent to tafamidis meglumine 80 mg).  ^b^Total annual rate of CV-related hospitalisations among all patients was calculated as: total CV-related hospitalisations across all patients divided by total years of study participation across all patients. CV relatedness was based on clinical evaluation and algorithmic assessment of FMQ names and preferred terms.  ^c^Risk ratio and *p*-value were derived using a Poisson regression model.  CI, confidence interval; CV, cardiovascular; FMQ, US Food and Drug Administration medical query; LTE, long-term extension study; NAC, National Amyloidosis Centre. | | | | | | |
