# Supplementary material for: Long‐term efficacy of tafamidis in patients with transthyretin amyloid cardiomyopathy by National Amyloidosis Centre stage
Source: Eur J Heart Fail. 2025 Jun 9;27(12):2998–3009. doi: 10.1002/ejhf.3696 (PMC12803551; doi:10.1002/ejhf.3696)
Supplement: Supplementary file 8 — Table S5. Baseline patient characteristics across expanded National Amyloidosis Centre (NAC) stages I–IV. [file EJHF-27-2998-s003.docx]

| **Table S5. Baseline patient characteristics across expanded NAC stages I–IV** | | | | | | | | |
| --- | --- | --- | --- | --- | --- | --- | --- | --- |
|  | **NAC Stage I** | | **NAC Stage II** | | **NAC Stage III** | | **NAC Stage IV** | |
|  | **Placebo to tafamidis**  ***n* = 71** | **Continuous tafamidis  80 mg**  ***n* = 75** | **Placebo to tafamidis**  ***n* = 69** | **Continuous tafamidis 80 mg**  ***n* = 58** | **Placebo to tafamidis**  ***n* = 29** | **Continuous tafamidis**  **80 mg**  ***n* = 32** | **Placebo to tafamidis**  ***n* = 8** | **Continuous tafamidis**  **80 mg**  ***n* = 8** |
| **Age, mean (SD), y** | 72.1 (7.4) | 73.2 (7.4) | 75.0 (5.4) | 75.3 (7.0) | 76.2 (5.6) | 79.5 (6.1) | 75.8 (10.2) | 76.0 (6.0) |
| **Sex, n (%)** |  |  |  |  |  |  |  |  |
| Male | 62 (87.3) | 69 (92.0) | 64 (92.8) | 54 (93.1) | 26 (89.7) | 24 (75.0) | 5 (62.5) | 8 (100.0) |
| Female | 9 (12.7) | 6 (8.0) | 5 (7.2) | 4 (6.9) | 3 (10.3) | 8 (25.0) | 3 (37.5) | 0 |
| **Race, n (%)** |  |  |  |  |  |  |  |  |
| White | 61 (85.9) | 59 (78.7) | 59 (85.5) | 50 (86.2) | 22 (75.9) | 18 (56.3) | 4 (50.0) | 6 (75.0) |
| Black | 9 (12.7) | 10 (13.3) | 9 (13.0) | 4 (6.9) | 6 (20.7) | 10 (31.3) | 2 (25.0) | 2 (25.0) |
| Asian | 1 (1.4) | 5 (6.7) | 1 (1.4) | 4 (6.9) | 1 (3.4) | 2 (6.3) | 2 (25.0) | 0 |
| American Indian or Alaska Native | 0 | 1 (1.3) | 0 | 0 | 0 | 2 (6.3) | 0 | 0 |
| ***TTR* genotype, n (%)** |  |  |  |  |  |  |  |  |
| Wild-type | 19 (26.8) | 16 (21.3) | 14 (20.3) | 10 (17.2) | 7 (24.1) | 14 (43.8) | 3 (37.5) | 2 (25.0) |
| Variant | 52 (73.2) | 59 (78.7) | 55 (79.7) | 48 (82.8) | 22 (75.9) | 18 (56.3) | 5 (62.5) | 6 (75.0) |
| **NT-proBNP,** **mean (SD), ng/L** | 1837.3 (665.5) | 1762.7 (694.3) | 4118.6 (1797.5) | 4303.4 (1534.7) | 5320.3 (1733.1) | 5895.8 (2008.5) | 13966.1 (2540.7) | 13542.4 (4171.2) |
| **mBMI, mean (SD)** | 1094 (197.7) | 1089 (173.7) | 1021 (182.0) | 1042 (159.2) | 1122 (194.8) | 1045 (186.4) | 1018 (206.2) | 1022 (178.4) |
| **eGFR,** **mean (SD), ml/min/1.73m2^b^** | 66.0 (13.4) | 67.8 (13.2) | 55.0 (14.3) | 56.1 (12.2) | 36.1 (6.3) | 34.9 (6.5) | 38.4 (11.3) | 43.6 (13.0) |
| **Troponin I, mean (SD), ng/mL** | 0.1 (0.2) | 0.1 (0.1) | 0.2 (0.2)^c^ | 0.2 (0.1) | 0.2 (0.1) | 0.7 (2.2) | 0.3 (0.2) | 0.3 (0.2) |
| **NYHA class, n (%)** |  |  |  |  |  |  |  |  |
| I | 6 (8.5) | 11 (14.7) | 6 (8.7) | 3 (5.2) | 1 (3.4) | 1 (3.1) | 0 | 0 |
| II | 48 (67.6) | 53 (70.7) | 37 (53.6) | 36 (62.1) | 12 (41.4) | 13 (40.6) | 4 (50.0) | 3 (37.5) |
| III | 17 (23.9) | 11 (14.7) | 26 (37.7) | 19 (32.8) | 16 (55.2) | 18 (56.3) | 4 (50.0) | 5 (62.5) |
| **Follow-up duration, median (95% CI), months**^d^ | 64.2 (57.5–77.8) | 63.3 (59.9–68.8) | 59.1 (51.6–81.5) | 63.3 (58.1–76.0) | 53.4 (29.7–NE) | 51.6 (30.6–60.9) | NE (NE–NE) | 54.5 (30.4–NE) |
| ^a^mBMI was calculated as the serum albumin level (g/L) multiplied by the BMI (weight in kg/square of the height in metres).  ^b^eGFR was estimated using the Chronic Kidney Disease Epidemiology Collaboration (CKD-EPI) equation.  ^c^*n* = 68.  ^d^Calculated using the Kaplan–Meier method.  BMI, body mass index; CI, confidence interval; eGFR, estimated glomerular filtration rate; mBMI, modified BMI; NAC, National Amyloidosis Centre; NE, non-estimable; NT-proBNP, N-terminal pro-B-type natriuretic peptide; SD, standard deviation; *TTR*, transthyretin. | | | | | | | | |
