# Supplementary material for: Long‐term efficacy of tafamidis in patients with transthyretin amyloid cardiomyopathy by National Amyloidosis Centre stage
Source: Eur J Heart Fail. 2025 Jun 9;27(12):2998–3009. doi: 10.1002/ejhf.3696 (PMC12803551; doi:10.1002/ejhf.3696)
Supplement: Supplementary file 9 — Data S1. Supporting Information. [file EJHF-27-2998-s009.docx]

# Supplemental methods

## Sensitivity analysis

The Tafamidis in Transthyretin Cardiomyopathy Clinical Trial (ATTR-ACT) was not designed to separate findings by dose. A sensitivity analysis was conducted for all-cause mortality, cardiovascular (CV)-related mortality, and CV-related hospitalisations in patients treated continuously with either tafamidis meglumine 80/20 mg (pooled, *n* = 261) throughout ATTR-ACT and the long-term extension study (LTE) vs. those treated with placebo in ATTR-ACT and then tafamidis in the LTE (*n* = 177). CV-relatedness was based on an evaluation and clinical judgment of Medical Dictionary for Regulatory Activities (MedDRA) preferred terms (*Table S1*). All-cause or CV-related mortality between treatment groups was assessed by a Cox proportional hazards model adjusted for treatment and baseline *TTR* genotype (wild-type or variant) with a two-sided maximum likelihood *p*-value and visualised using Kaplan–Meier method. Heart transplant and cardiac mechanical assist device (CMAD) implantation were treated as death events in the mortality assessments. Relative risk ratio and *p*-value for CV-related hospitalisation were derived using a Poisson regression model. The total annual rate of CV-related hospitalisations among all patients was calculated as: (total number of CV-related hospitalisation across all participants) / (total years of study participation across all participants). CV-related hospitalisations per patient per year was calculated as: (patient’s number of CV-related hospitalisations) / (duration on study [years]).

## Supplementary analysis

Using the expanded National Amyloidosis Centre (NAC) staging system, we performed a supplementary analysis of all-cause and CV-related mortality in patients receiving either continuous tafamidis 80 mg or continuous tafamidis 80/20 mg (pooled) vs. placebo to tafamidis across baseline NAC stages I–IV.^8^ All-cause and CV-related mortality and CV-related hospitalisations were assessed using the same methodology as described for the sensitivity analysis.
